# Efficacy and Safety of Peroral Endoscopic Myotomy in the Treatment of Zenker’s Diverticulum: A Single-Center Experience

**DOI:** 10.5152/tjg.2024.23402

**Published:** 2024-02-01

**Authors:** Abdullah Murat Buyruk, Çağdaş Erdoğan

**Affiliations:** 1 Department of Gastroenterology, Ege University School of Medicine, İzmir, Turkey; 2 Department of Gastroenterology, University of Health Sciences, Ankara Etlik City Hospital, Ankara, Turkey

**Keywords:** Zenker’s diverticulum, Zenker’s diverticulum peroral endoscopic myotomy, endoscopic treatment, myotomy, endoscopy

## Abstract

**Background/Aims:**

The efficacy and safety of Zenker’s peroral endoscopic myotomy (Z-POEM), a current method in the treatment of Zenker’s diverticulum (ZD), have been demonstrated in a limited number of studies and case reports. This study aimed to report our experience with the Z-POEM method.

**Materials and Methods:**

Patients with ZD who were treated with Z-POEM between January 2019 and March 2023 and had a follow-up period of at least 3 months were included in the study. Our primary endpoint was clinical success. A Kothari–Haber score (KHS) of 2 or less at 1 month postoperatively was defined as clinical success. Our secondary endpoints were adverse events and recurrence rates.

**Results:**

In total, 20 patients (males, 65%; mean age, 63 ± 14.4 years) were treated with Z-POEM. The mean ZD septum length was 33.7 (±11.04) mm. The technical success rate was 100% (20/20), and the clinical success rate was 95% (19/20). In 1 case with a large ZD (septum length of 60 mm), the mucosal septum, which was thought to cause partial persistence of symptoms, was treated by endoscopic septotomy. The mean KHS decreased significantly after Z-POEM (preoperative KHS: 7.3 and postoperative KHS: 0.15, *P* < .0001). The median follow-up period was 10 months (interquartile range, 3-39). No recurrence was observed in any case. Intraprocedural mild subcutaneous emphysema was observed in 4 (20%) cases. Emphysema regressed spontaneously in the postoperative period without any treatment.

**Conclusion:**

Zenker’s peroral endoscopic myotomy is a successful and reliable method in the treatment of ZD, with low recurrence rates.

**Main Points**

- Zenker’s diverticulum is a pulsion diverticulum, and surgical and endoscopic techniques can be used in its treatment.
- The Zenker’s peroral endoscopic myotomy (Z-POEM) method is one of the endoscopic techniques, is an effective and safe method based on cricopharyngeal myotomy while preserving the esophageal mucosa.
- In our study, it was aimed to evaluate the efficacy and safety of the Z-POEM method, and the Z-POEM procedure was applied to a total of 20 patients, with 100% technical success and 95% (19/20) clinical success.
- Our study is the largest Z-POEM series performed in Turkey and contributes to the literature at this point.

## Introduction

Zenker’s diverticulum (ZD), also known as hypopharyngeal diverticulum, is a type of pulsion diverticulum first described in 1877.^1^ It occurs when the hypopharyngeal mucosa and submucosa herniate into the Killian triangle (the triangle between the transverse fibers of the cricopharyngeal muscle at the bottom and the fibers of the inferior pharyngeal constrictor muscle at the top).^2^ Its prevalence rate is 0.01%-0.11%.^3^ It is most commonly observed in older men (aged >70 years) and is usually asymptomatic.^4^ The most common symptoms are dysphagia, regurgitation, cough, hoarseness, halitosis, weight loss, and pneumonia. The etiopathogenesis of ZD is unknown. It has been suggested that the increase in intraluminal pressure caused by dyscoordination of oral, pharyngeal, and esophageal muscles during swallowing facilitates the development of a defect in the pharyngeal wall and causes herniation.^5^

There are 3 main types of treatment modalities for ZD: surgery, rigid endoscopic treatments, and flexible endoscopic treatments. Because of high complication rates, endoscopic treatments have replaced surgical treatments. Among endoscopic treatments, flexible endoscopic treatments are preferred because of the lower risk of adverse events and the absence of the need for neck hyperextension in rigid endoscopic procedures. Flexible endoscopic septotomy (FES) is the most commonly used flexible endoscopic treatment method.^6^ FES has high clinical success (56%-100%), but the high risk of perforation (6.5%) and recurrence rate (11%-30%) are its negative aspects.^7-9^ In 2016, Li et al^10^ modified the peroral endoscopic myotomy (POEM) method originally developed for the treatment of cardia achalasia for the management of ZD. Initially termed “submucosal tunneling endoscopic septum division,” this method was later designated as Zenker’s peroral endoscopic myotomy (Z-POEM) by Hernandéz Mondragón et al^11^ in 2018. The Z-POEM method aims to better expose the cricopharyngeal muscle through submucosal tunnels on both sides of the septum, resulting in a more complete myotomy. By protecting the overlying mucosa during the procedure, the risk of leakage and mediastinitis in the postoperative period is reduced.^10^ The efficacy (84%-95.8%) and safety (rate of adverse events, 4.9%-20.8%) of Z-POEM, a current treatment, have been demonstrated in a small number of studies with a limited number of cases.^9,12-19^ The aim of our single-center study was to evaluate the efficacy and safety of Z-POEM in the treatment of ZD.

## Materials and Methods

### Study Population

In this single-center retrospective cohort study, data from 20 patients with ZD treated with Z-POEM at Ege University Hospital between January 2019 and March 2023 were recorded. Patients with a follow-up period of less than 30 days were excluded from the study. The diagnosis of ZD was made by barium esophageal radiography and confirmed by endoscopic examination. The length of the septum between the ZD and the esophageal lumen was calculated by measurement on barium radiography.

Demographic data, symptoms on presentation, previous treatments for ZD (if any), details of the Z-POEM procedure, adverse events, duration of hospitalization, and presence of recurrence were recorded. The comorbid conditions of the patients were scored according to the Charlson Comorbidity Index (CCI).^20^ The Kothari–Haber score (KHS) was used to evaluate symptoms. This score includes 6 symptoms in addition to dysphagia: regurgitation, cough, hoarseness, halitosis, weight loss, and pneumonia. The KHS score ranges between 0 and 16 points according to the frequency or severity of symptoms.^21^

The study protocol was approved by the Ethics Committee of Ege University (approval date: March 23, 2023, approval number: 23-3.1T/14). Informed consent was obtained from all patients regarding the Z-POEM procedure.

### Zenker’s Peroral Endoscopic Myotomy Procedure

All patients adhered to the clear diet list for the last 5 days before Z-POEM. All patients were hospitalized in the morning on the day of the procedure. All procedures were performed in the operating room under general anesthesia, with the patient in a supine position. All procedures were performed by a single endoscopist. All patients received a prophylactic dose of 1 g ceftriaxone (intravenously) 30 minutes before Z-POEM. Carbon dioxide gas was used during the procedures. Before Z-POEM, the diverticulum pouch and esophageal lumen were washed with a 0.9% saline solution to remove residual debris. Before initiating Z-POEM, a guidewire was left in the stomach to stabilize the septum.

**Figure 1.**
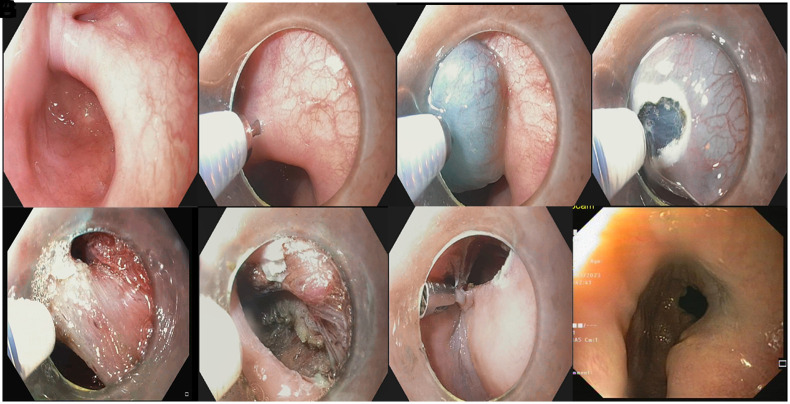
Steps of Z-POEM: (A) Zenker’s diverticulum, (B, C) mucosal injection, (D) mucosal incision, (E) cricopharyngeal muscle was isolated from the mucosal side, (F) muscular septotomy, (G) mucosal closure, (H) third month endoscopic view.

The modified technique described by Mavrogenis et al^22^ was used for all Z-POEM procedures. This method consisted of 4 stages (Figure 1A-H). In the first stage, the submucosal solution mixture (3-5 mL) consisting of saline + methylene blue was injected into the submucosa on the septum with a sclerotherapy needle, and a linear mucosal incision of 10-15 mm was made in the mucosa, which swelled in the form of a hill after the injection. In the second stage, 2 separate submucosal tunnels were opened on the esophageal and diverticular sides. Submucosal tunnels exposed the cricopharyngeal muscle, which was isolated from the mucosal side. In the third stage, a cricopharyngeal myotomy extending 1 cm to the esophageal side was performed. After myotomy, the lumen was washed with a mixture of gentamicin 80 mg + saline (10 mL). Before the clips were applied, an endoscopy from the hypopharynx to the esophagus was performed to check for residual resistance. If no resistance or dragging was felt, the mucosectomy was closed with clips. After the procedure, the esophageal mucosa was checked for perforation. The successful realization of all stages was defined as technical success.

The ST Hood (Fujifilm, Tokyo, Japan) was used in all procedures. A 3-mm flush knife (Fujifilm, Tokyo, Japan) was used for mucosal incision and submucosal tunnel opening. During myotomy, a 3.5-mm clutch cutter (Fujifilm, Tokyo, Japan), a triangle-type knife (KD-640L; Olympus, Japan), or a 3-mm flush knife (Fujifilm, Tokyo, Japan) was used. The ESG 400 (Olympus, Japan) electrocautery device was used in all procedures. Pulse cut slow (effect 2/40 watt) mode was applied during mucosectomy; spray COA (effect 2/30 watt) mode was applied during submucosal tunneling; and pulse cut slow (effect 2/40 watt) and spray COA modes (effect 2/30 watt) were used in combination during myotomy. Soft coagulation (effect 3/60 watt) was applied with monopolar hemostatic forceps (FD-410 LR, Olympus) in cases of bleeding that could not be stopped with the knife. Acetaminophen 1 g and tramadol 100 mg were administered as perioperative analgesics.

### Postoperative Follow-up

Oral feeding was not allowed for the first 8 hours after Z-POEM. Patients adhered to the clear diet for the first 3 days and then consumed soft foods for the next 10 days. Normal feeding was resumed after the second week. No patient had a control esophagogram before enteral feeding.

Pain scoring using a visual analog score was performed at the fourth postoperative hour and the first postoperative day by the primary physician who performed the procedure. Accordingly, pain intensity was evaluated as 0 = no pain, 1-3 = mild pain, 4-6 = moderate pain, 7-9 = severe pain, and 10 = most severe pain.

In the postoperative period, ceftriaxone and metronidazole (intravenous) antibiotic treatments were administered on the first day. Amoxicillin/clavulanate suspension (5 days) and sucralfate treatments were started as maintenance, and the patients were discharged. At the time of discharge, patients received the contact number of the doctor who performed the procedure in case of any complaints.

Patients were evaluated in the outpatient clinic at the first, third, sixth, and twelfth postoperative months to monitor any recurrence of symptoms.

### Outcome Measures

The primary endpoint of the study was the clinical and technical success of Z-POEM. Technical success was defined as the successful completion of the above-mentioned stages of the Z-POEM procedure. A KHS of ≤2 at the 1-month follow-up was defined as clinical success.

Secondary endpoints were Z-POEM-related adverse events and recurrence rates.

### Statistical Analysis

The Statistical Package for the Social Sciences Statistics software, version 26.0 (IBM Inc., Armonk, NY, USA) was used to conduct the statistical analysis. For categorical data, a number and a percentage were provided. Continuous variables that were proven to be normally dispersed were given as mean and standard deviation. Otherwise, the median and interquartile range were used. For nonparametric variables, Spearman’s correlation analysis was used.

## Results

In this study, 20 patients (mean age 63 ± 14.4 years) were included, with 65% being male. The average septum length was 33.7 (±11.04) mm, and preprocedural KHS averaged 7.3 (±4.2), while CCI averaged 2.6 (±2.1). Almost all patients were treatment-naive, except 1 (5%). The Z-POEM procedure achieved technical success in all patients (20/20, 100%) with an average duration of 36.1 (±13.4) minutes. Various tools [a clutch cutter (Fujifilm, Tokyo, Japan) was used in 3 cases, a triangle knife (KD-640L; Olympus, Japan)] were used during the myotomy procedure, and mucosectomy closure required a median of 5 clips (IQR, 4-6).

Postprocedural KHS significantly improved (mean 0.15 ± 0.4; *P* < .001), with clinical success observed in 95% of patients. One patient with persistent symptoms had a giant ZD with a 60-mm-long septum. This patient, who had a preoperative KHS of 16, showed a significant clinical improvement after Z-POEM, and his KHS decreased to 5 in the first postoperative month. A control endoscopy performed 3 months after the procedure revealed that the mucosa on the septum, which was previously subjected to cricopharyngeal myotomy, was causing the current complaints. The mucosal septum was treated by endoscopic septotomy. At the sixth postoperative month, the KHS was 0, the patient gained 18 kg, and complaints regressed completely. Minor subcutaneous emphysema occurred in 20% of cases but resolved spontaneously. There were no postoperative complications. Pain scores were low (Visual Analogue Scale [VAS] 2.1 ± 1.5 at 4 hours and 0.5 ± 0.6 at 1 day postoperatively). Hospitalization averaged 1.3 (±0.4) days.

The median follow-up period was 10 (IQR, 3-39) months. There was no worsening of symptoms in any patient during follow-up (Table 1).

## Discussion

In the present investigation, we have methodically showcased both the effectiveness and safety of the modified Z-POEM procedure within the confines of a tertiary health-care institution. Furthermore, it is noteworthy that this study represents the most extensive case series conducted to date within the Turkish medical landscape. Zenker’s diverticulum is a pulsion diverticulum, and it can be treated with surgical and endoscopic methods. Zenker’s POEM method is one of the endoscopic techniques, is an effective and safe method based on cricopharyngeal myotomy while preserving the esophageal mucosa. There is not much research on Z-POEM’s effectiveness and safety in our nation. Our study assessed 20 patients who received Z-POEM; the technical success rate was 100% (20/20), and the clinical success rate was 95% (19/20). Short-term (median follow-up <12 months) follow-up results of Z-POEM, a current method in the treatment of ZD, are promising.^9,12-17^ According to the results of a meta-analysis including 196 cases from 9 retrospective studies, the clinical success rate of Z-POEM was 93.4%, the frequency of adverse events was 4.9%, and the recurrence rate was 6.6%.^18^ In a multicenter, international, retrospective study conducted by Steinway et al,^19^ Z-POEM was shown to be a successful (clinical success rate, 94%) and safe (frequency of adverse events, 8.9%; recurrence rate, 6.7%) treatment in the long term (median follow-up, 37 months). According to the European Society of Gastrointestinal Endoscopy Guidelines, Z-POEM is currently considered an experimental therapy in the treatment of ZD and is recommended to be applied within a research setting for the time being.^23^

Our study is important in terms of having the largest Z-POEM series reported from Turkey. The Z-POEM method was successfully completed in all patients. Clinical success was achieved in all but 1 patient. In the patient without clinical success, it was observed that the mucosa on the septum, which was 60-mm long before treatment, led to the continuation of the symptoms by forming a pouch after Z-POEM. This case was treated with the FES method. There is only 1 study showing that the clinical success rate of Z-POEM in large ZDs (≥40 mm) is relatively low (85%).^24^ In large ZDs, mucosal septotomy during Z-POEM may reduce recurrence in these cases.^7^ However, prospective randomized studies are needed. Mild subcutaneous emphysema was observed in 4 patients during Z-POEM. However, as in POEM, it is controversial whether submucosal emphysema should be considered an adverse event in Z-POEM.

In Z-POEM treatment, it is difficult to open a submucosal tunnel and apply clips to the mucosectomy site because of the stenosis of the working area in the hypopharynx.^10^ In the modified Z-POEM method, it has been shown that performing mucosectomy directly over the septum instead of the hypopharynx facilitates treatment and shortens the procedure time.^15,19,22^ No significant difference has been reported in terms of recurrence or adverse events with respect to the mucosectomy site.^9^ In our series, mucosectomies were performed over the septum in all patients, and the mean procedure time was 36.1 minutes. The shorter procedure time in the present study compared to the conventional Z-POEM series may be related to the modified Z-POEM method. Considering that Z-POEM is a procedure that requires technical skill, the experience of the endoscopist should also be considered.

In the 2022 study conducted by Al Ghamdi et al,^17^ a comparative analysis was undertaken between Z-POEM and septotomy, revealing that their respective clinical success rates were closely aligned. Z-POEM exhibited a marginally higher success rate. In a study conducted by Swei et al^25^ in 2023, a similar comparison was made between Z-POEM and septotomy. Septotomy exhibited a clinical success rate of 86.7%, whereas Z-POEM achieved a 100% success rate. Recurrence rates were observed to be comparable in both approaches. Upon scrutinizing the content of these studies, it was observed that the average diverticulum size was consistent at 2.4 ± 0.6 in both investigations. In our study, clinical success was achieved in 95% of cases (19 out of 20). However, there was 1 exceptional case where a patient with a substantial Zenker’s diverticulum, featuring a 60-mm-long septum, did not achieve clinical success. While this patient initially experienced symptom improvement, complaints related to the mucosal septum resurfaced during follow-up, necessitating a subsequent septotomy. This occurrence prompts us to consider that as the size of the septum increases, a larger portion of the mucosa remains, potentially leading to the persistence of symptoms. Therefore, it could prove beneficial to take the size of the septum into account when selecting the appropriate treatment approach.

There is only 1 study in the literature on the efficacy of Z-POEM in recurrent ZD, and according to this study, Z-POEM is highly effective (96.7%) in recurrent ZD after surgical or endoscopic procedures.^26^ In the present study, only 1 case had a history of previous treatment (endoscopic stapler septotomy). This case was successfully treated with the Z-POEM method.

In the literature, more than 1 scoring system has been used in the evaluation of response to treatment after Z-POEM. The Dakak and Benett scoring system, which is the most used scoring system, evaluates only dysphagia, while the KHS evaluates other symptoms such as regurgitation and cough.^9,21^ One of the strengths of the present study is that the Z-POEM treatment response was evaluated using the KHS. A common classification system is needed to compare the clinical success of different studies on ZD.

The present study and most studies in the literature include cases reported by tertiary centers. Therefore, this limits the generalizability of the results for less experienced centers. The small number of patients in the present study is another limitation. However, the number of patients in the present study was similar to that of the limited studies on Z-POEM in the literature. Considering that ZD is a rare condition, we believe that our case series will increase the sample size for future studies and contribute to the evidence of the efficacy and safety of Z-POEM. Another limitation of the present study is the short follow-up period of the patients. In our patient group, only 7 cases had a follow-up period of >1 year. This significantly hinders our ability to comment on the long-term recurrence rates after Z-POEM. However, given that Z-POEM has been available in Turkey for the past 4 years, future studies will address this issue as follow-up periods extend.

The results obtained in the present study showed that Z-POEM is effective and safe for the treatment of ZD. Zenker’s peroral endoscopic myotomy is also a promising method in terms of short-term recurrence.

## Figures and Tables

**Table 1.** Demographic and Clinical Characteristics of Patients who Underwent Zenker’s Peroral Endoscopic Myotomy

		*P*
Age, mean, years	63 ± 14.4	
Gender, male, %, n	65%, (13)	
Septum length, mean, mm	33.7 (±11.04)	
Presenting symptoms, %, n Dysphagia Regurgitation Cough Hoarseness Halitosis Weight loss Pneumonia	100% (20) 90.0% (18) 60.0% (12) 60.0% (12) 45.0% (9) 45.0% (9) 20.0% (4)	
CCI, mean	2.6 (±2.1)	
Previous treatments, %, n Surgical diverticulectomy Naive	5.0% (1) 95.0% (19)	
Preprocedural KHS, mean	7.3 (±4.2)	
Postprocedural KHS, mean	0.15 (±0.4)	**<.001**
Technical success, %, n	100% (20)	
Clinical success, %, n	95.0% (19)	
Procedure duration, mean, minutes	36.1 (±13.4)	
Complications, %, n Subcutaneous emphysema	20.0% (4)	
Hospital stay, mean	1.3 (±0.4)	
Follow-up, median, month	10 (IQR, 3-39)	

CCI, Charlson Comorbidity Index; IQR, interquartile range; KHS, Kothari–Haber score.
